# HIV and the gut microbiome: future research hotspots and trends

**DOI:** 10.3389/fmicb.2025.1466419

**Published:** 2025-02-07

**Authors:** Zhen Wu, Zhan-Peng Xie, Xin-Xin Cui, Xiang-Bin Sun, Fang-Yi Zhao, Nuo Wang, Yu Li, Haixia Wang, Li Zhang, Jing Shen, Fulei Chen, Haogang Sun, Jia He

**Affiliations:** ^1^Medical School of Shihezi University, Shihezi, China; ^2^Department of Preventive Medicine, Medical School of Shihezi University, Shihezi, China; ^3^Key Laboratory for Prevention and Control of Emerging Infectious Diseases and Public Health Security, The Xinjiang Production and Construction Corps, Ürümqi, China; ^4^School of Medicine, First Affiliated Hospital, Shihezi University, Shihezi, China

**Keywords:** HIV/AIDS, gut microbiome, bibliometrics, short-chain fatty acids, T cells, obesity

## Abstract

**Background:**

The use of highly active antiretroviral therapy has transformed AIDS into a chronic infectious disease, but issues of chronic inflammation and immune system activation persist. Modulating the gut microbiome of patients may improve this situation, yet the specific association mechanisms between HIV and the gut microbiome remain unclear. This study aims to explore the research hotspots and trends of the HIV and the gut microbiome, providing direction for future research.

**Methods:**

We conducted a search of the Web of Science Core Collection database up to April 30, 2024 to retrieve articles related to the relationship between the HIV and the gut microbiome. The scientific achievements and research frontiers in this field were analyzed using CiteSpace, VOSviewer, and Bibliometrix statistical software.

**Results:**

As of April 30, 2024, a total of 379 articles met the inclusion criteria. The number of publications in this field peaked in 2023, and the number of articles published after 2020 declined. The country with the highest number of publications was the United States (184 articles), and the institution with the most publications was the University of Colorado (USA) (21 articles). The author with the most publications was Routy Jean-Pierre (Canada) (14 articles). High-frequency keywords, aside from the key terms, included “HIV,” “inflammation,” “immune activation,” “gut microbiota,” and “translocation.” Keyword burst results indicated that short-chain fatty acids, T cells and obesity might become the focus of future research.

**Conclusion:**

The research hotspots in this field should prioritize examining the role of the primary gut microbiome metabolite, short-chain fatty acids, in reducing immune system activation and inflammation. Another emerging area of interest could be the investigation into the annual increase in obesity rates within this field. Furthermore, understanding the metabolic mechanisms of short-chain fatty acids in T cells is essential. Additionally, multi-omics analysis holds potential.

## 1 Introduction

Acquired immune deficiency syndrome (AIDS) is caused by HIV infection. It is a chronic infectious disease, which can lead to immune deficiency (Chen and Lai, 2023; du et al., 2023). HIV primarily targets CD4+ T cells, compromising immune function and leading to the development of opportunistic infections and death (Sponaugle et al., 2023; Wang et al., 2023). HAART has increased CD4+ T cell counts in people living with HIV (PLWH), thereby extending their lifespans (Wen et al., 2023). However, approximately 15–30% of patients experience an inability to restore CD4+ T cell counts, termed immunological non-responders (Yang et al., 2020). CD4+ T cell destruction is not the sole factor in HIV progression; it is influenced by multifaceted factors, with sustained immune activation being the most significant, largely driven by gut microbiome dysbiosis (Rb-Silva et al., 2019; Nakanjako et al., 2016; Moretti et al., 2023). Alzahrani et al. (2019) demonstrated that supplementing probiotics to PLWH can reduce abnormal immune system activation and inflammatory responses, thereby improving HIV progression. Additionally, 16S sequencing reveals differences in gut microbiome between PLWH and healthy individuals (Zhang et al., 2023). However, the specific mechanism underlying gut microbiome dysbiosis in PLWH remains unclear. Therefore, investigating research hotspots and trends in HIV and the gut microbiome holds promise for addressing issues such as sustained immune activation and gut microbiome dysbiosis in the progression of HIV/AIDS in this population.

Bibliometrics is a quantitative method for visualizing scientific information that demonstrates research trends and hotspots in a specific field (Fu et al., 2023). To date, no reports on bibliometric studies of the gut microbiome and HIV exist. In this study, we utilized Citespace 6.1.R6, VOSviewer 1.6.19, and Bibliometrix to analyze literature on “HIV and the gut microbiome” published up to April 30, 2024. Our aim was to identify changes in hotspots and trends, providing directions for the future prevention and treatment of HIV.

## 2 Materials and methods

### 2.1 Database resources

The Web of Science Core Collection (WoSCC, Clarivate Analytics, Philadelphia, PA, USA) was chosen for literature retrieval in this study. The search included articles from the establishment of the database to April 30, 2024.

### 2.2 Search strategy

To capture as many articles as possible, this study used the MeSH terms from the PubMed database. The search strategy for this study was created following the retrieval rules of WoSCC, using the main subject terms “HIV/AIDS and gut microbiome” along with associated keywords (specific search strategy available in the Supplementary material).

### 2.3 Literature screening and inclusion criteria

This study included research papers and review articles as the types of literature. The search was limited to publications in the English language. Two independent researchers conducted the screening process, resulting in a total of 2,372 records retrieved. By examining the titles, abstracts, and keywords of the retrieved literature, the researchers determined whether they had clear relevance to both “gut microbiome” and “HIV.” Manual filtering was performed, and if the relevance could not be determined solely based on the title, abstract, and keywords, the full text was consulted. Duplicate records and articles that clearly did not meet the inclusion criteria were excluded. Each researcher identified 353 and 401 articles, respectively. The results of the included analysis were cross-checked, and any disagreements regarding inclusion were resolved by a third researcher. Ultimately, 1,994 articles were excluded, and a total of 379 articles met the inclusion criteria. The flowchart of the study process is presented in Supplementary Figure 1.

### 2.4 Analytical methods

A total of 379 articles that met the inclusion criteria were selected for this study. These articles were exported and downloaded in Plain Text File, Tab Delimited File, and BibTeX formats. We used CiteSpace version 6.1.R6 (Drexel University, Pfizer, America) to extract and organize burst keywords, overlay dual maps of journals, and generate keyword timeline graphs based on their frequency of occurrence. Additionally, co-occurrence maps were generated. VOSviewer version 1.6.18 (Leiden University, Leiden, Netherlands) was utilized to identify the countries, institutions, journals, and keywords associated with the articles, and to create co-occurrence maps. The bibliometrix package in R Studio version 4.2.3 was employed for visualizing the analysis of countries and authors. Microsoft Office Excel 2019 (Microsoft, America) was used for data management and analysis of publication trends, including yearly articles and co-citations.

## 3 Results

### 3.1 Publication output and journals

**Figure 1:**
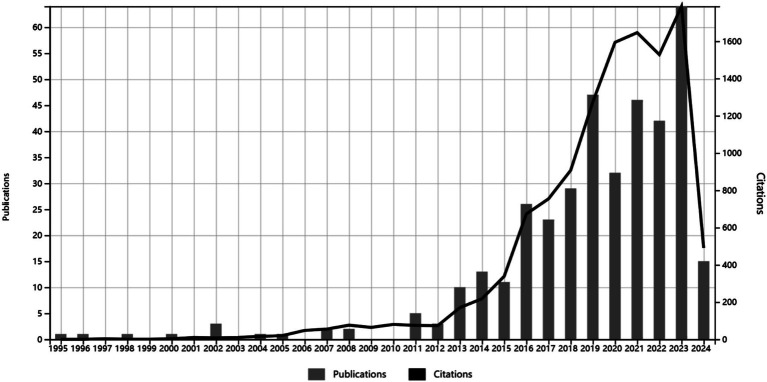
The volume of publications and citations fluctuates with the passage of time.

A total of 379 articles were included based on the search strategy and selection criteria, covering the period from January 1, 1995, to April 30, 2024. The articles’ output and their co-citation network are depicted in Figure 1. From 1995 to 2012, there was relatively low publication output, with a peak of 5 articles in 2011. The volume of publications surged from 2013 to 2019, indicating widespread interest in research on the relationship between gut microbiome and HIV. The year 2023 marked the peak of publication output, followed by a decline in articles after 2020. Articles related to gut microbiome and HIV were published in 149 different journals, as shown in Supplementary Figure 2. The top 10 journals are listed in Supplementary Table 1, with MICROBIOME having the highest impact factor and the AIDS having the highest publication output. Furthermore, we conducted a dual-map overlay of journals for HIV and gut microbiome, as shown in Supplementary Figure 3, to further explore the thematic distribution of journals and the transfer path of interdisciplinary knowledge. The cited journals mainly belong to clinical, molecular biology, and medical disciplines, while the citing journals are primarily from molecular, biological, and genetic fields.

### 3.2 Visualization analysis of authors, countries, and institutions collaboration

**Figure 2:**
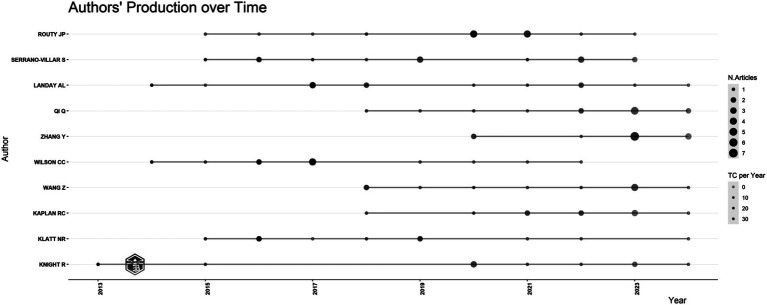
The quantity of articles written by the author fluctuates over time.

As depicted in Figure 2, the top 10 authors in terms of publication volume include Routy Jean-Pierre, who has published 14 papers, ranking first (seven articles, seven reviews). Routy Jean-Pierre’s research agenda is predominantly centered on intervention methodologies encompassing probiotics, cannabinoids, Metformin, and terbinafine, within the framework of HIV and the gut microbiome (Costiniuk et al., 2019; Isnard et al., 2020; el-Far et al., 2021; Ouyang et al., 2023). Notably, all publications by these authors have surfaced within the last decade.

**Figure 3:**
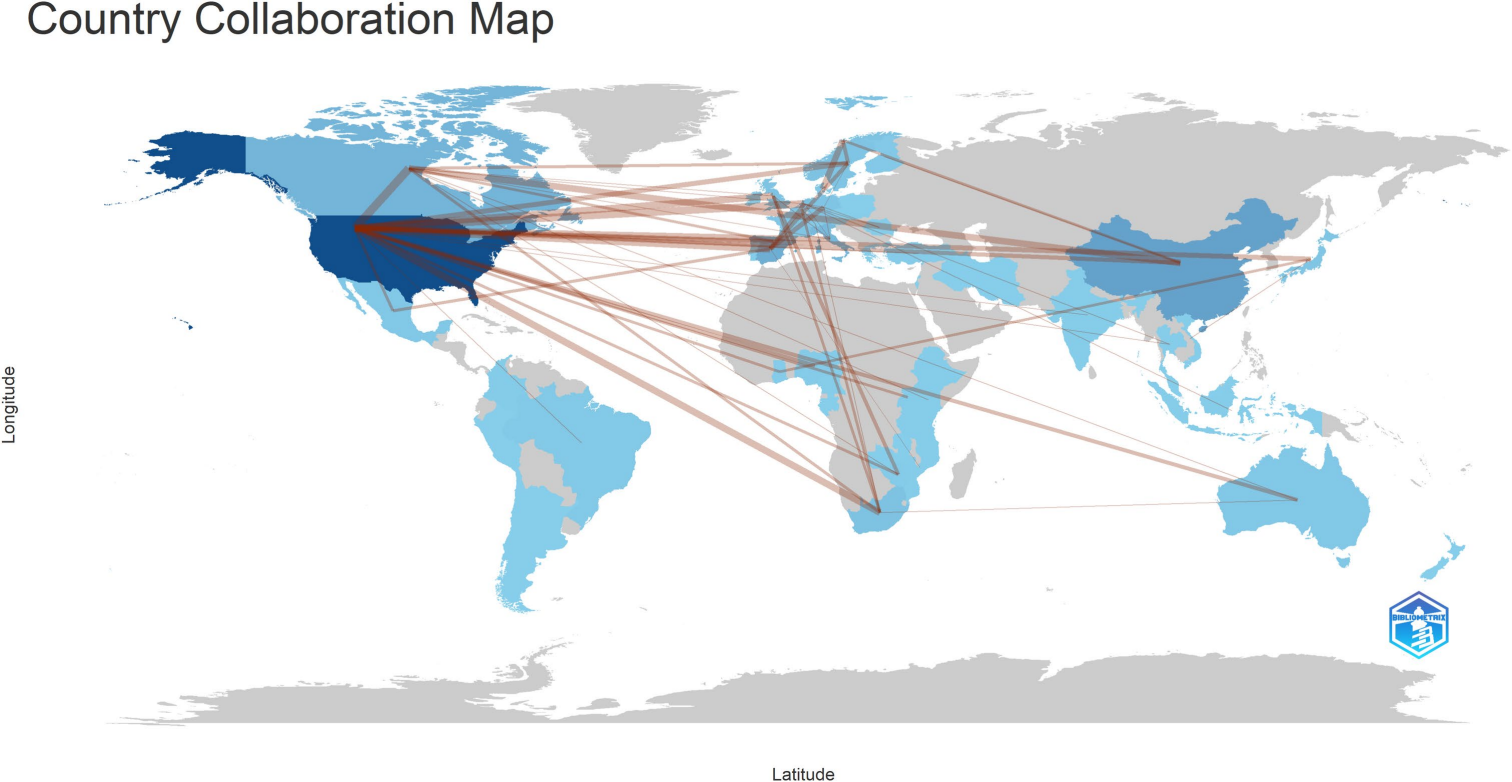
The visualization of national output collaboration. With darker colors representing higher output and thicker lines indicating closer collaboration.

A total of 51 countries are engaged in research on HIV and gut microbiome, visualized in Figure 3. The United States has the highest number of publications, with 184 papers, followed by China with 59. Thirteen countries have published more than 10 papers. Collaboration among countries is primarily driven by high-output nations, with significant interactions among them. The country outbreak graph is shown in Supplementary Figure 4, revealing that the primary publishing countries in the future will be Ghana, Belgium, Switzerland and Nigerla with two of these being developing nations.

**Figure 4:**
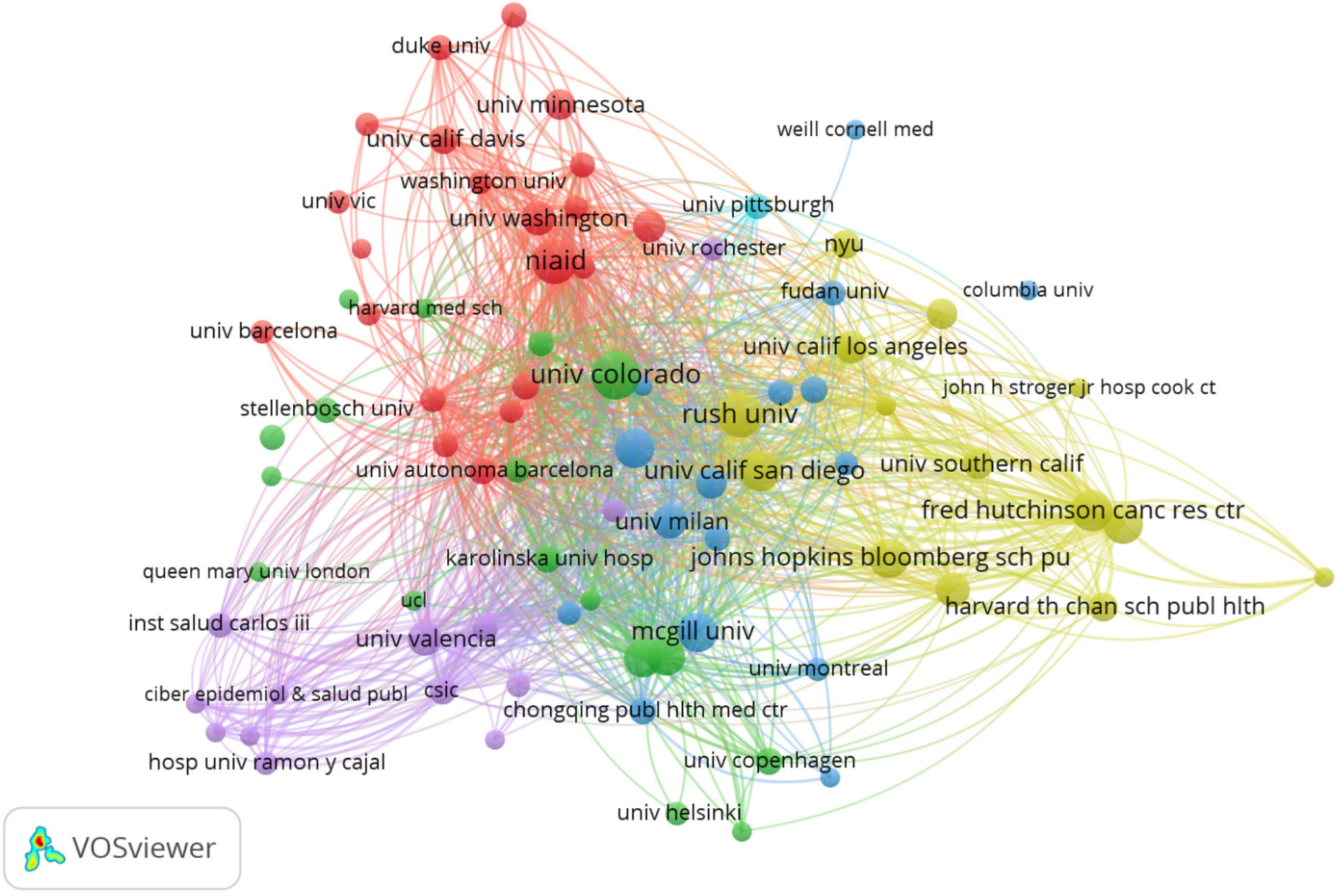
The institution produces visual analyses. Where the size of the nodes represents the volume of publications, and the thickness of the connections represents the level of collaboration.

A total of 817 institutions have published papers, as depicted in the visualization in Figure 4. The University of Colorado is the leading institution in terms of publication volume, with 21 papers. The top 10 institutions are predominantly from developed countries, with eight based in the United States, indicating the leading position of the United States in this field. The institution outbreak graph is shown in Supplementary Figure 5, with Capital Medical University being the sole institution from a developing country.

### 3.3 Analysis of article citations

The top 10 cited articles are presented in Supplementary Table 2. Among them, nine were authored by institutions based in the United States. Eight of these articles concentrate on 16S sequencing and genome sequencing and investigate differences in gut bacterial and viral populations among PLWH, non-HIV-infected individuals, and PLWH under HAART treatment (Vujkovic-Cvijin et al., 2013; Dillon et al., 2014; Dinh et al., 2015; Lozupone et al., 2013; Mutlu et al., 2014; Monaco et al., 2016; Noguera-Julian et al., 2016; Handley et al., 2012). These investigations did not incorporate proteomics or metabolomics methodologies, and the sample sizes were relatively modest, with only one study encompassing over 100 participants and a total sample size of 240 (Noguera-Julian et al., 2016). Please refer to Supplementary Figure 6 for a comprehensive overview of the seminal reference. Vujkovic-Cvijin and Somsouk (2019) deliver a thorough review delineating the mechanisms underlying alterations in the gut microbiome during HIV disease progression, while also proposing intervention strategies for enhancing gut microbiome health. Furthermore, they investigate the influence of lifestyle and behavioral factors on the microbiome. Additionally, they explored through cohort studies that the dysbiosis of the microbiome in PLWH is unrelated to gender and sexual behavior, but rather correlates with nadir CD4 levels, inflammatory markers, and comorbidities (Vujkovic-Cvijin et al., 2020). Armstrong et al. (2018) discussed the factors contributing to intestinal flora imbalance in individuals with AIDS.

### 3.4 Keyword analysis

**Figure 5:**
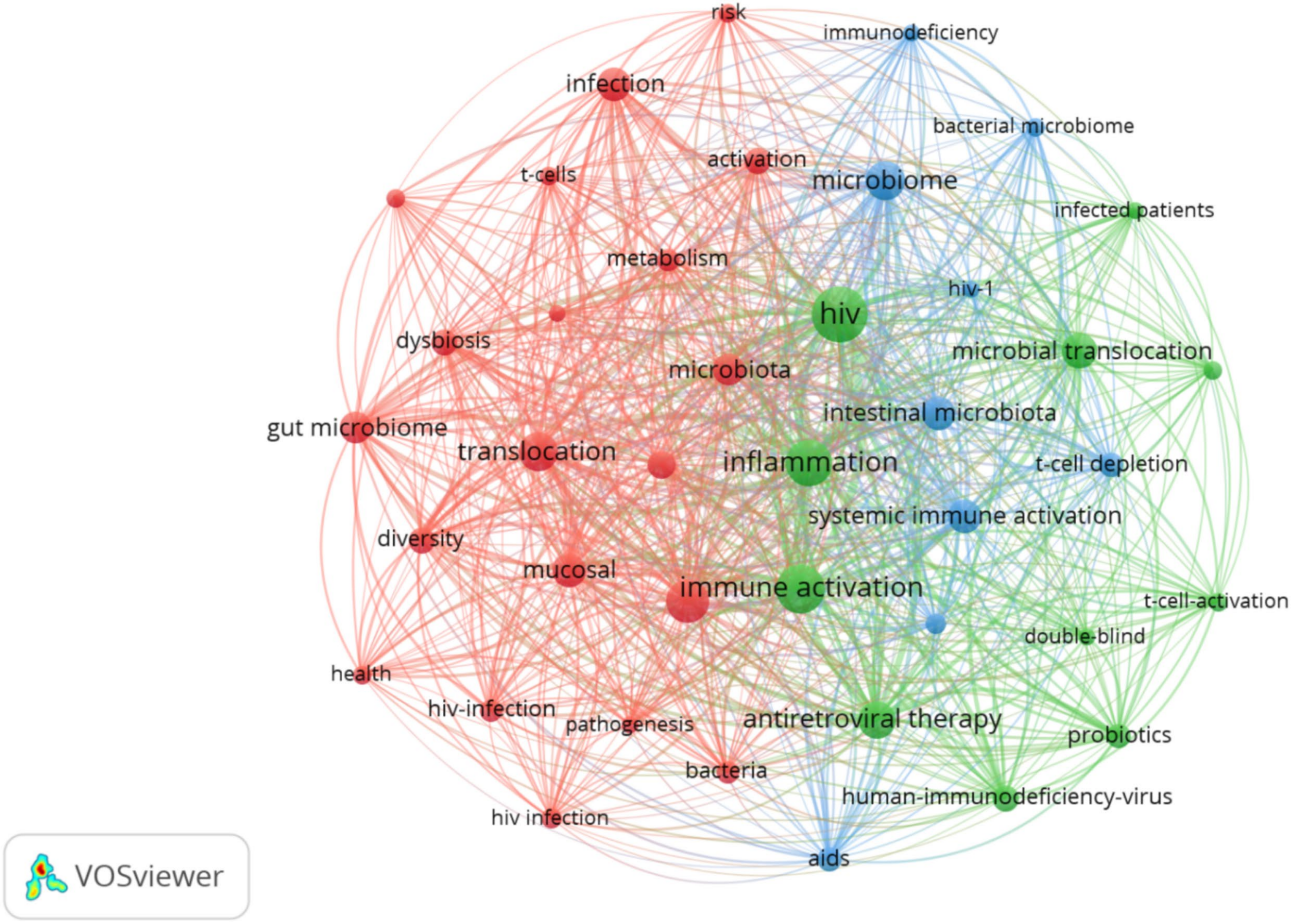
Keyword visual analysis. Node size represents keyword frequency, thickness represents the strength of connection.

A total of 1,712 keywords were included and incorporated into the keyword network, as shown in Figure 5. Among these, inflammation, immune activation and translocation were the most frequently occurring and closely interconnected keywords, Clustering analysis primarily focused on three domains: first (Red clustering), The mechanism of microbial dysbiosis in PLWH; second (Green clustering), interventions in this field (Blue clustering); Clinical manifestations and classification in this field. The 20 most significant keywords in this domain are presented in Supplementary Figure 7. The earliest appearance is associated with microbiome, spanning from 2007 to 2013, with an intensity rating of 2.49. Future research should continue to pay attention to short-chain fatty acids, T cells, and obesity.

## 4 Discussion

### 4.1 General information

The fluctuation in the volume of academic publications serves as a crucial indicator for the development trend of a field. Prior to 2019, the publication volume exhibited a yearly increasing trend, while after 2019, the publication volume began to decline. This timing coincides with the outbreak of COVID-19, leading to speculation that the global research focus shifted toward the prevention and treatment of COVID-19, consequently resulting in fewer studies on HIV and other infectious diseases. The decrease in PLWH^1^ may also be related to the Joint United Nations Programme on HIV/AIDS target of zero new PLWH by 2030 (Xue et al., 2023), which may have affected the publication volume on HIV and gut microbiome. However, with the end of the pandemic and the ongoing publication of longitudinal research papers, the number of articles in this field is expected to rise. As a result, there is anticipation for additional clarification on the mechanisms that underlie the relationship between gut microbiome and HIV. At the same time, we found that the number of articles published in this field began to increase in 2013, and we also found that nine of the top 10 cited articles were published after 2012. The research of Handley et al. (2012) found that enterovirus is the main reason for HIV progress through genome analysis. Later, various studies studied the difference and role of flora in PLWH and healthy people through 16 s sequencing and genome sequencing. In addition, The United Nations Programme on HIV/AIDS said in 2014 that 90% of people living with HIV should reduce the viral load to zero, while 10–40% of PLWH have poor immune cell recovery after receiving highly effective antiretroviral reduction disease, which becomes poor immune reconstitution, and intestinal flora plays an important role in this mechanism (Wan et al., 2023). Therefore, we believe that the above two reasons are the reasons for the increase in publication volume in 2013. Analysis of the visualized journal citation network revealed that the AIDS and Frontiers in Immunology were the top journals for publication volume, while Microbiome has the highest impact factor, thus providing high-quality frontier information for HIV and gut microbiome research.

The visualization of countries, institutions, and authors indicates that the publication volume and influence of developed countries far surpass those of developing countries for HIV and gut microbiome research. However, compared to developed countries, there are inconsistencies in terms of race, dietary habits, lifestyle customs, and the misuse of antibiotics in developing countries (Batool et al., 2023). Parbie et al. (2021) utilized high-throughput macroeconomic tools for the first time to characterize the fecal microbiome of HIV-1-infected Ghanaian adults. A Dutch cohort was selected as a representative non-African cohort for comparison. The HIV- and HIV+ groups in their study exhibited significantly lower alpha diversity and significantly different beta diversity compared to their Dutch counterparts (Parbie et al., 2021). As a result, research findings from developed countries may not be applicable to developing countries. In terms of the number of PLWH, developing countries greatly exceed developed countries, with sub-Saharan Africa (SSA) accounting for two-thirds of the total (Sadler et al., 2023). Burkhart Colorado et al. discovered that, in comparison to urban areas in Zimbabwe, successful antiretroviral therapy (ART) in rural PLWH exhibited inferior outcomes in terms of reducing intestinal microbial composition and T-cell activation (Burkhart Colorado et al., 2024). This article, published in Microbiome, suggests that there is considerable potential for research in HIV and gut flora related research in SSA.

Institutions and countries that rank high in publication volume demonstrated closer interconnections, suggesting that cooperation leads to more fruitful results. Hence, developing countries should actively collaborate with developed countries to jointly explore the specific mechanisms of gut microbiome and HIV for the cure of HIV or immunological reconstruction for HIV. The top five institutions in terms of publication volume are all from the United States, with very few research institutions from developing countries, particularly from SSA. However, observations from the visualized outbreak maps of countries and institutions indicate that future publications from developing countries and institutions will gradually increase. In Figure 2, Routy Jean-Pierre emerges as the most prolific contributor, as evidenced by the highest publication volume. His scholarly endeavors center around the investigation of influential determinants and intervention strategies within the designated field. Initially, his research delved into examining the correlation between PLWH and alterations in the gut microbiome, positing a potential linkage to immune activation and T cell dynamics (Lu et al., 2018). Subsequently, adopting a gut microbiome-centric approach, he explored various intervention modalities, encompassing probiotics, metformin interventions, and terbinafine interventions (Costiniuk et al., 2019; Isnard et al., 2020; el-Far et al., 2021; Ouyang et al., 2023; Lu et al., 2018).

### 4.2 Research hotspots

Keywords can indicate the evolution and research hotspots in this field. Based on Figures 5 and Supplementary Figure 7. Future research endeavors are anticipated to predominantly revolve around three focal areas: fundamental inquiries, intervention methodologies, and the exploration of adverse repercussions. Insights gleaned from keyword burst analyses suggest a burgeoning interest in topics such as short-chain fatty acids, T cells, and obesity, signaling a progressive trajectory. These areas hold promising potential as viable treatment modalities for HIV, underscoring their significance in advancing therapeutic strategies.

Current research indicates that the disruption of the gut microbiome caused by HIV had been confirmed (Li et al., 2024). The dysregulation of the gut microbiome leading to immune system activation and chronic inflammation is an important factor in the development of HIV (Munjoma et al., 2023). However there have been few reports on the metabolic products of the gut microbiome and the metabolic pathways in the progression and pathogenesis of HIV. Existing studies on the metabolic products of the gut microbiome indicate that probiotics can produce short-chain fatty acids (SCFAs) (e.g., butyrate) which can induce regulatory T cells to reduce immune activation and alleviate gut inflammatory responses thus slowing down the progression of HIV (Enriquez et al., 2023). Furthermore they can also promote the production of CD4+ T cells interleukin-10 and interleukin-22 maintaining gut immunity and promoting immune reconstitution in the body (Kibbie et al., 2021). The dysregulation of the gut microbiome elicited by HIV encompasses a diminution in beneficial bacteria alongside an augmentation of pathogenic counterparts. Concurrently it fosters intestinal barrier impairment exacerbating immune system activation and precipitating dysregulated immune responses. This immune system activation precipitates a perturbation in immune homeostasis. Moreover the dysregulation engenders an imbalance in the differentiation between Treg and Th17 cells precipitating an elevation in pro-inflammatory cytokines coupled with a reduction in anti-inflammatory counterparts thereby instigating chronic inflammation.

Notably, short-chain fatty acids play a pivotal role in ameliorating intestinal barrier integrity and bolstering anti-inflammatory cytokine levels (Sereti et al., 2023). The gut microbiome and its metabolites hold potential sway over the development and differentiation of bone marrow cells and B cells, potentially influencing aberrant antibody differentiation (Geng et al., 2020). Jiang et al. (2023) corroborate the notion that short-chain fatty acids can enhance the specific immune response elicited by HIV vaccines, aligning with their immunomodulatory role and capacity to foster antibody responses. However, there is limited research on medium-chain and long-chain fatty acids in relation to HIV. In addition to their anti-inflammatory properties, long chain fatty acid esters significantly inhibit the proliferation and cytokine secretion of CD4 + and CD8 + T cells. Treatment of T cells with mitochondrial targeted antioxidant mito tembo can restore T cell function damage caused by eicosahexaenoic acid. Therefore, inhibiting the action of long-chain fatty acids can restore T cell function damage (Li et al., 2023). Furthermore, the majority of research on SCFAs had been cross-sectional, indicating the need for large-scale prospective studies to elucidate the specific mechanisms and causal relationships of the metabolic products of the gut microbiome in the development of HIV. While the mechanism of action of SCFAs in HIV is not yet clear, it is currently possible to increase the content of SCFAs by supplementing beneficial bacteria to improve the gut microbiome (Ma et al., 2023).

As depicted in Figures 5. Probiotics and prebiotics are important intervention measures for increasing SCFAs and restoring the stability of the gut microbiome. The results of the random effects meta-analysis showed that probiotic supplementation resulted in a moderate increase in CD4 lymphocyte counts in ART-untreated individuals. In contrast, no significant changes were observed in ART-experienced individuals (Oyadiran et al., 2024; Sachdeva et al., 2022). However, if probiotics are used as an adjunctive treatment for HIV, they could help to improve PLWH’s life Quality. Studies have shown that the use of probiotics may be therapeutically helpful in preventing diarrhea and re-establishing normal gut microbiota in PLWH (Irvine et al., 2010; Carter et al., 2016). Subgroup analyses conducted for intervention time showed that the incidence of AIDS-related diarrhea decreased when probiotics were given for more than 30 days. When probiotics were given for <30 days, there was no change in the incidence of AIDS-related diarrhea. Where the type and geographical location of the probiotic did not affect the results (Zhang et al., 2021). This hints to researchers to design experiments on the effectiveness of probiotic-assisted HIV treatment with adequate intervention times. Of course, other confounding factors such as probiotic dose and treatment modality (monobacteria or mixed bacteria), mode of administration, and diet need to be taken into account. In conclusion, we emphasize that probiotics can only be used as adjunctive therapy in the treatment of HIV and that conducting large-scale cohort studies over a long period is strong evidence to validate the efficacy of probiotic-assisted treatment of HIV. We look forward to the exploration and additions of future researchers in this direction.

T cells had become a hot topic in this field, as the HIV infects cells expressing the CD4 molecule, such as CD4+ T cells, macrophages, and dendritic cells. The gut contains a large number of CD4+ T cells and macrophages, serving as a reservoir for the HIV. Therefore, the elimination of infected cells in the gut is crucial for the treatment of HIV. Shytaj, IL and others had found that down- regulation of glycolysis is a metabolic feature of the latent period of HIV-infected cells. It is still not clear what role glycolysis plays in the latency of the virus (Shytaj et al., 2021). Studies had shown an increase in glycolysis in immune cells during infection, and Zhang, JH and others had found that blocking glycolysis can inhibit the M1 polarization of macrophages, which is associated with chronic inflammation (Zhang et al., 2023). Furthermore, bacterial lipopolysaccharides can promote the activation of immune cells and the production of pro-inflammatory factors, while SCFAs in the gut microbiome can be metabolized by immune cells, providing anti-inflammatory effects by supplying energy to immune cells and inhibiting glycolysis. In the future, immunometabolism may become a treatment for HIV. Researchers should focus on the metabolic effects of gut microbiome metabolites in immune cells.

The incidence of obesity among PLWH is progressively rising, a trend attributed to several factors. Firstly, the utilization of HAART has extended the lifespan of PLWH, consequently aligning their obesity rates with those of the general populace (Cook et al., 2020). Secondly, research by Baltazar-Díaz et al. suggests that different HAART medications exert varying effects on the gut microbiota, thereby influencing weight gain differentially (Baltazar-Díaz et al., 2023). Furthermore, obese PLWH individuals are prone to immune cell activation and heightened production of inflammatory cytokines like IL-6. The confluence of obesity and HIV fosters dysbiosis of the intestinal flora, exacerbating both AIDS and obesity—a phenomenon perpetuating a deleterious cycle necessitating prompt intervention. However, findings from Gogokhia et al. contend that irrespective of the HAART regimen employed, the obesity incidence consistently rises. Consequently (Gogokhia et al., 2020), the etiology likely encompasses multifaceted factors, including lifestyle choices. Future investigations should delve into elucidating the specific determinants to inform targeted interventions effectively.

### 4.3 Research trends

Upon analysis of the top 10 cited articles, it is noted that the sample size in these studies is generally small, with limited capacity to control confounding factors. Currently, a large amount of literature pertains to cross-sectional studies on the gut microbiome and HIV. Future efforts should address the specific causal relationships and mechanisms through longitudinal studies, as well as expanding the sample size to obtain more reliable results. In terms of research methodology, 16S sequencing and genomic studies are predominant, while metabolomics, proteomics, and transcriptomics are less prevalent. Genomic studies often focus on the classification differences of the microbiome, lacking functional information, which can be complemented by metabolomics and proteomics. Metabolomics can evaluate the role of gut microbiome metabolites, opening new avenues for the prevention and treatment of HIV. Proteomics can use SCFAs as a starting point to investigate their effects on PLWH, exploring the pathways and mechanisms of the gut microbiome in HIV.

The efficacy of the HIV eradication strategy hinges upon a comprehensive comprehension of HIV-1 infected cells, which exhibit significant heterogeneity. The advent of single-cell multi-group methodologies offers a solution to address the challenges posed by this heterogeneity (Wong et al., 2023). For instance, Wu et al. used a single-cell assay for transposase accessible chromatin (scATACseq) and employed a human-virus comparison strategy developed in a chimeric antigen receptor (CAR) T-cell model, incorporating the presence of integrated provirus as a molecular beacon to identify PLWH-infected cells from treated and untreated PLWH directly (Wu et al., 2023). They provided the first direct and unbiased identification of experimentally and *in vivo* infected memory CD4 T cells at single-cell resolution, thereby accelerating our understanding of HIV reservoirs to target therapies. Multi-omic studies of the microbiome can provide insight into the mechanisms underlying host–microbe interactions (Chetty and Blekhman, 2024). Bailin et al. used multiomics factor analysis (MOFA) results to suggest that SAT dysregulation and dyslipidemia were major contributors to phenotypic variation in metrics associated with cardiometabolic disease in a contemporary ART virus-suppressed PLWH cohort (Bailin et al., 2024). A relatively small number of multi-omics studies have been conducted in the area of HIV and gut flora. This may be related to the high cost of sample collection and data generation.

## 5 Conclusion

In this study, we employed CiteSpace, VOSviewer, and Bibliometrix to evaluate 379 articles focusing on the research of HIV and the gut microbiome up to April 30, 2024. Our analysis reveals a burgeoning field, with contributions emanating from 51 countries, 817 institutions, and involving 1,712 authors. Moving forward, we recommend longitudinal research for sustained observational insights, emphasizing the exploration of causal mechanisms linking the gut microbiome and HIV, particularly in relation to T cells and chain fatty acids. It is recommended that researchers explore the potential of probiotics as an adjunctive treatment for HIV. Additionally, it is imperative to investigate the underlying factors driving the increasing incidence of obesity and implement targeted intervention measures. Furthermore, we advocate for supplementing our understanding of gut microbiome metabolites through multi-omics analyses, with the aim of unveiling novel avenues for the prevention and treatment of HIV.

## Data Availability

The original contributions presented in the study are included in the article/Supplementary material, further inquiries can be directed to the corresponding author.

## Footnotes

^1^ https://www.unaids.org/en/resources/fact-sheet
