# Supplementary material for: HIV and the gut microbiome: future research hotspots and trends
Source: Front Microbiol. 2025 Feb 7;16:1466419. doi: 10.3389/fmicb.2025.1466419 (PMC11844347; doi:10.3389/fmicb.2025.1466419)
Supplement: Supplementary file 1 [file Supplementary_file_1.docx]

**HIV and the gut microbiome: Future research hotspots and trends.**

**Appendix 1**

**HIV and the gut microbiome term**

（ hiv or AIDS or Immunologic Deficiency Syndrome, Acquired or Acquired Immune Deficiency Syndrome or Acquired Immuno-Deficiency Syndrome or Acquired Immuno Deficiency Syndrome or Acquired Immuno-Deficiency Syndromes or Immuno-Deficiency Syndrome, Acquired or Immuno-Deficiency Syndromes, Acquired or Syndrome, Acquired Immuno-Deficiency or Syndromes, Acquired Immuno-Deficiency or Immunodeficiency Syndrome, Acquired or Acquired Immunodeficiency Syndromes or Immunodeficiency Syndromes, Acquired or Syndrome, Acquired Immunodeficiency or Syndromes, Acquired Immunodeficiency or Human Immunodeficiency Virus or Immunodeficiency Virus, Human or Immunodeficiency Viruses, Human or Virus, Human Immunodeficiency or Viruses, Human Immunodeficiency or Human Immunodeficiency Viruses or Human T Cell Lymphotropic Virus Type III or Human T-Cell Lymphotropic Virus Type III or Human T-Cell Leukemia Virus Type III or Human T Cell Leukemia Virus Type III or LAV-HTLV-III or Lymphadenopathy-Associated Virus or Lymphadenopathy Associated Virus or Lymphadenopathy-Associated Viruses or Virus, Lymphadenopathy-Associated or Viruses, Lymphadenopathy-Associated or Human T Lymphotropic Virus Type III or Human T-Lymphotropic Virus Type III or AIDS Virus or AIDS Viruses or Virus, AIDS or Viruses, AIDS or Acquired Immune Deficiency Syndrome Virus or Acquired Immunodeficiency Syndrome Virus or HTLV-III ）AND (Gastrointestinal icrobiome OR Gastrointestinal Microbiomes OR Microbiome, Gastrointestinal OR Gut Microbiome OR Gut Microbiomes OR Microbiome, Gut OR Gut Microflora OR Microflora, Gut OR Gut Microbiota OR GutM Microbiotas OR Microbiota, Gut OR Gastrointestinal Flora OR Flora, Gastrointestinal OR Gut Flora OR Flora, Gut OR Gastrointestinal Microbiota OR Gastrointestinal Microbiotas OR Microbiota, Gastrointestinal OR Gastrointestinal Microbial Community OR Gastrointestinal Microbial Communities OR Microbial Community, Gastrointestinal OR Gastrointestinal Microflora OR Microflora, Gastrointestinal OR Gastric Microbiome OR Gastric Microbiomes OR Microbiome, Gastric OR Intestinal Microbiome OR Intestinal Microbiomes OR Microbiome, Intestinal OR Intestinal Microbiota OR Intestinal Microbiotas OR Microbiota, Intestinal OR Intestinal Microflora OR Microflora, Intestinal OR Intestinal Flora OR Flora, Intestinal OR Enteric Bacteria OR Bacteria, Enteric ）
