# Supplementary material for: HIV and the gut microbiome: future research hotspots and trends
Source: Front Microbiol. 2025 Feb 7;16:1466419. doi: 10.3389/fmicb.2025.1466419 (PMC11844347; doi:10.3389/fmicb.2025.1466419)
Supplement: Supplementary file 2 [file Supplementary_file_2.docx]

**HIV and the gut microbiome: Future research hotspots and trends.**

**Appendix 2**

**supplementary figure 1** **Retrieval process flowchart for the research** 2

**supplementary figure 2 Network visualization map of journal co-citation analysis generated by VOSviewer**. 3

**supplementary figure 3 The dual-map overlay of journals in intestinal flora and HIV** 4

**supplementary figure 4** **National outbreak words** 5

**supplementary figure 5 Agency outbreak words** 6

**supplementary figure 6 References outbreak** 7

**supplementary figure 7 Keyword line diagram** 8

**supplementary figure 8 Keywords outbreak** 9

**supplementary Table 1 Top 10 most productive journals** 10

**supplementary Table 2 Top 10 highly cited literature** 11

Publications identified through Web of Science database searching

(WoS Core Collection. SCI-Expanded)

1.Retrieval mode: Advanced Search

2.Retrieval strategy:

See attached table

3.Retrieval time: 2024.04.30

2073 publications were preliminarily identified for further screening

Retrieval time span: 1995.01.01-2024.04.30 Document types: Articles and Reviews

Language: (English)

Irrelevant literature was excluded by two independent authors by title, abstract and full-text screening

379 publications including 301 articles and 78 reviews were included in final analysis (Online platform,Bibliometrix.VOSviewer and CiteSpace)

Institutions/Authors

Countries/Regions

Journals

Research Areas

Keywords

###### **supplementary figure** 1：Retrieval process flowchart for the research


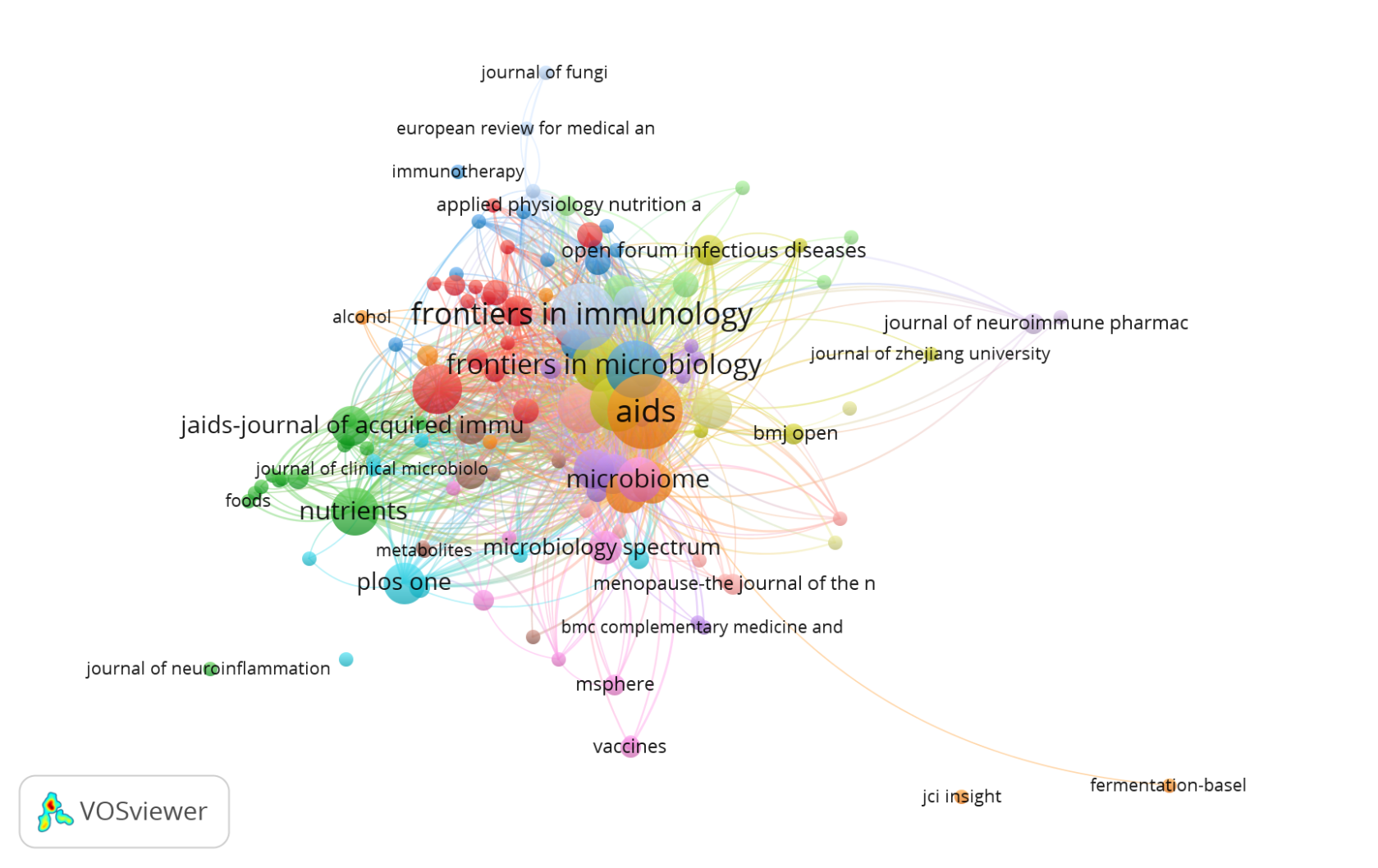


###### **supplementary figure** 2：Network visualization map of journal co-citation analysis generated by VOSviewer.


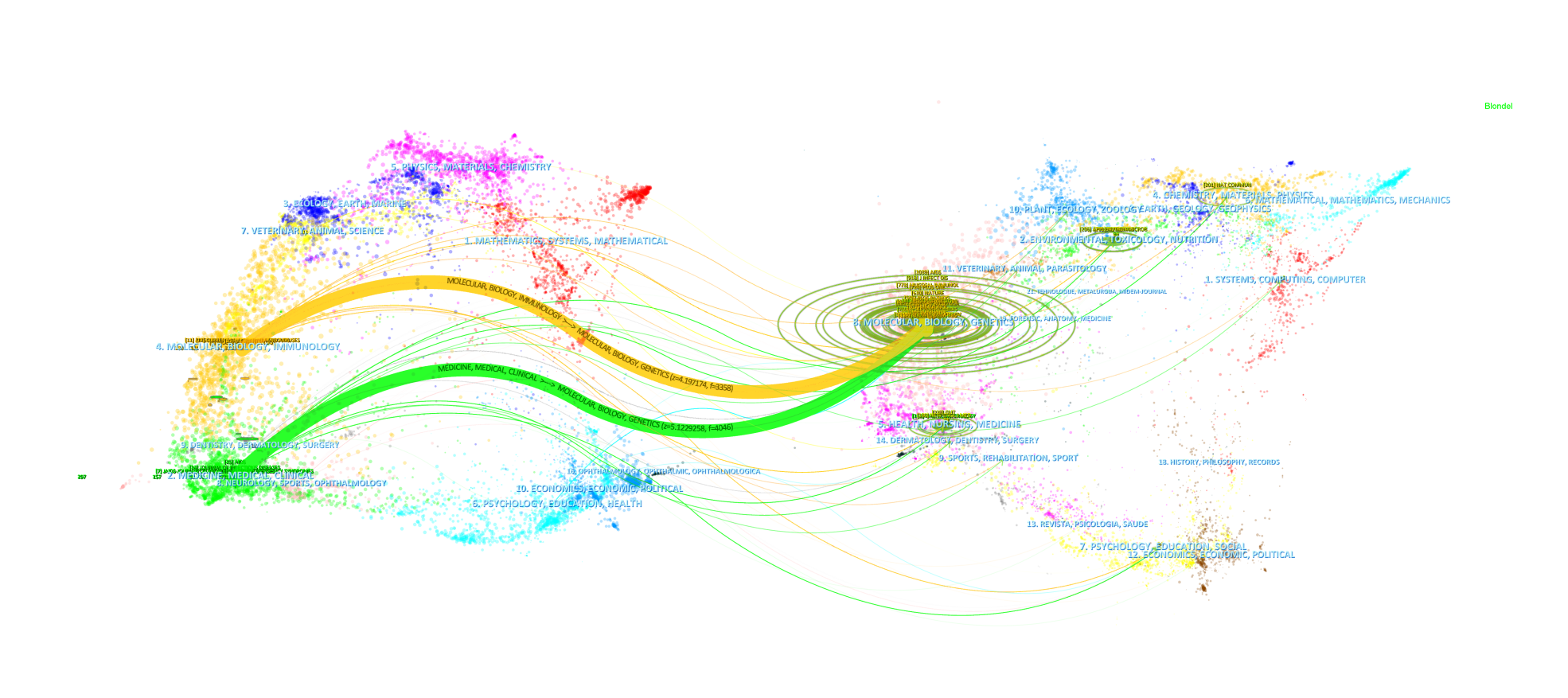


###### **supplementary figure** 3 : The dual-map overlay of journals in intestinal flora and HIV

Note: A dual-map overlay of journals shows the distribution of topics. The citing journals are on the left, and the cited journals are on the right. The labels represent the disciplines covered by the journals, and the colored path represents the citation relationship.


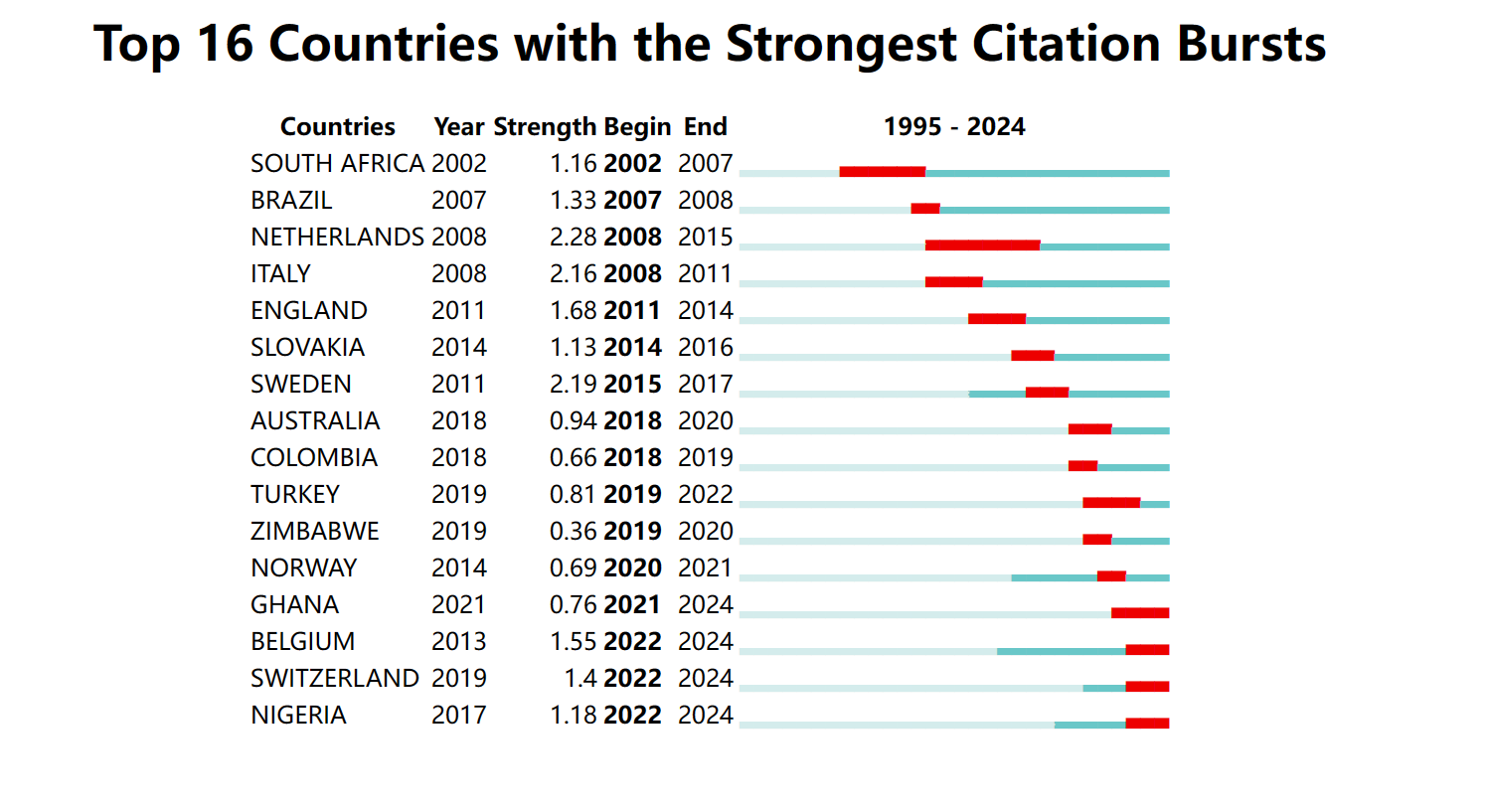


**supplementary figure 4** National outbreak words

Note:When the end is highlighted in red, it indicates that the country will be a primary publishing country in the future.


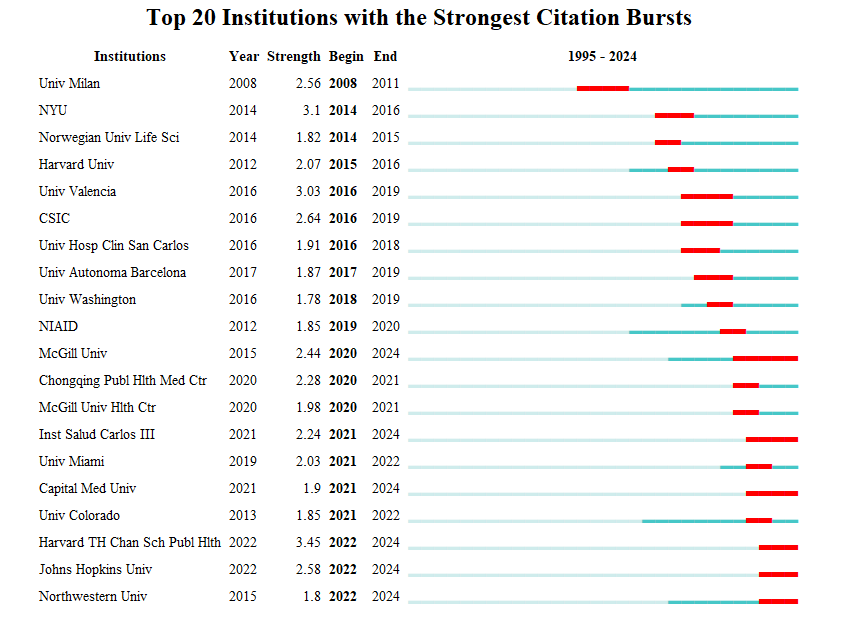


**supplementary figure 5** Agency outbreak words

Note:When the end is highlighted in red, it signifies that the institution will be a prominent publishing organization in the future.


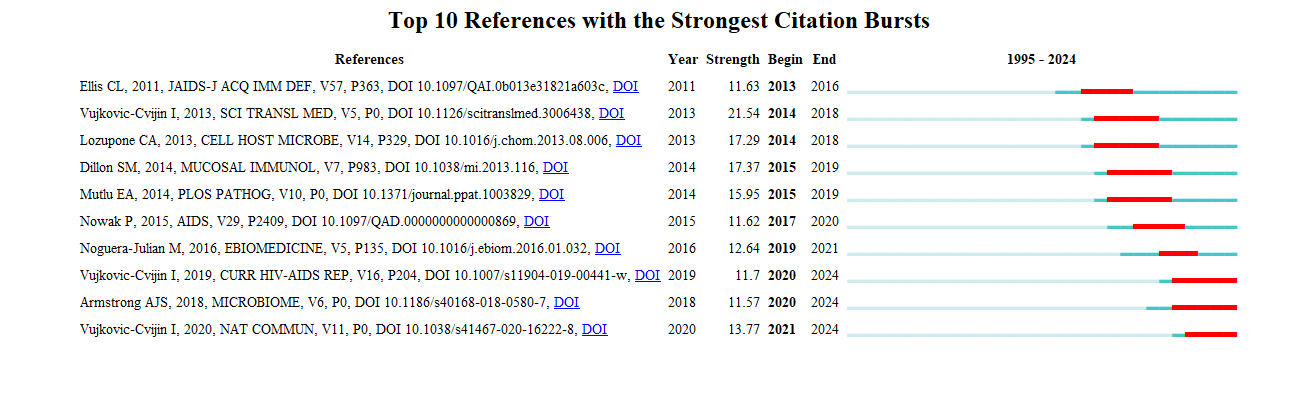


**supplementary figure 6** References outbreak

Note:When the end is highlighted in red, it indicates that the reference is expected to be one of the top-cited references in the future


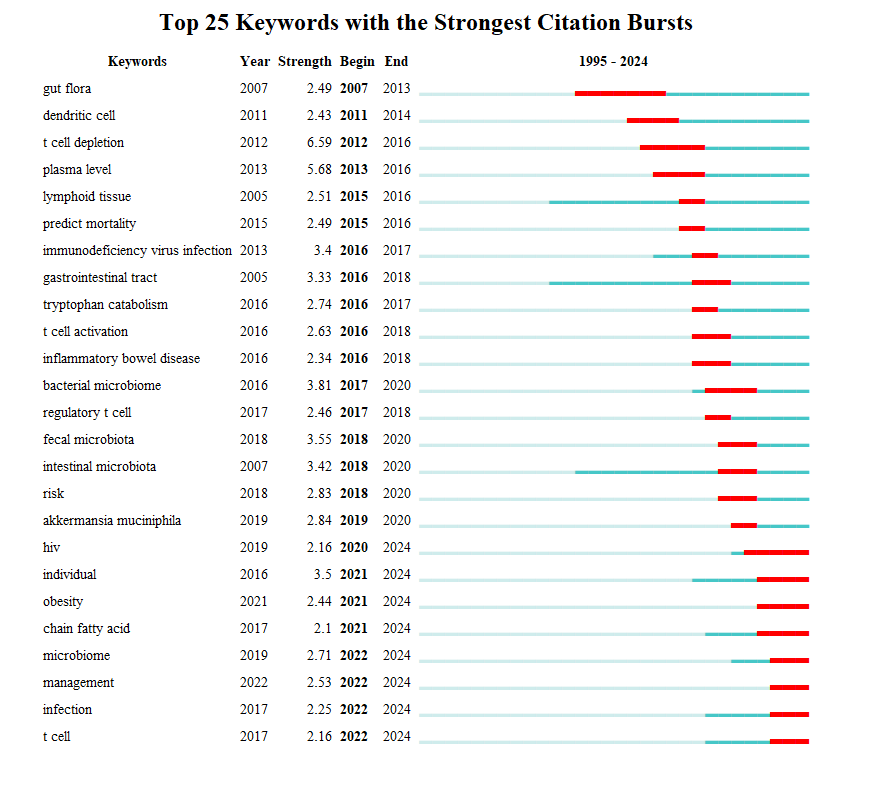


**supplementary figure 7** Keywords outbreak

Note:The red color at the end indicates that this keyword is the main research direction for the future

**supplementary Table 1：Top 10 most productive journals.**

| Ranking | Journal | Output | % of 379 | IF  2022 | JCR 2022 |
| --- | --- | --- | --- | --- | --- |
| 1 | AIDS | 25 | 6.596 | 3.90 | Q3 |
| 2 | Frontiers in Immunology | 20 | 5.227 | 7.30 | Q1 |
| 3 | Scientific Reports | 15 | 3.958 | 4,69 | Q2 |
| 4 | JOURNAL OF INFECTIOUS DISEASES | 14 | 3.694 | 6.40 | Q1 |
| 5 | Frontiers in Microbiology | 13 | 3.430 | 5.20 | Q2 |
| 6 | CURRENT OPINION IN HIV AND AIDS | 12 | 3.166 | 4.1 | Q3 |
| 7 | AIDS RESEARCH AND HUMAN RETROVIRUSES | 11 | 2.902 | 8.00 | Q1 |
| 8 | NUTRIENTS | 10 | 2.639 | 5.90 | Q1 |
| 9 | MICROBIOME | 9 | 2.375 | 15.5 | Q1 |
| 10 | EBIOMEDICINE | 8 | 2.111 | 11.1 | Q1 |

**supplementary Table 2：Top 10 highly cited literature.**

| Ranking | Title | First author | Year | Citations | Citations/Year |
| --- | --- | --- | --- | --- | --- |
| 1 | Dysbiosis of the Gut Microbiota Is Associated with HIV Disease Progression and Tryptophan Catabolism | [Vujkovic-Cvijin, I](https://webofscience.clarivate.cn/wos/author/record/39993226) | 2013 | 494 | 41.17 |
| 2 | Microbial Translocation in the Pathogenesis of HIV Infection and AIDS | [Marchetti, G](https://www.webofscience.com/wos/author/record/819950) | 2013 | 348 | 29.00 |
| 3 | An altered intestinal mucosal microbiome in HIV-1 infection is associated with mucosal and systemic immune activation and endotoxemia | Dillon, SM | 2014 | 345 | 31.46 |
| 4 | Perils at mucosal front lines for HIV and SIV and their hosts | Haase, AT | 2005 | 343 | 17.15 |
| 5 | Intestinal Microbiota, Microbial Translocation, and Systemic Inflammation in Chronic HIV Infection | [Dinh, DM](https://webofscience.clarivate.cn/wos/author/record/20742962) | 2015 | 332 | 33.20 |
| 6 | Alterations in the Gut Microbiota Associated with HIV-1 Infection | [Lozupone, CA](https://webofscience.clarivate.cn/wos/author/record/48184290) | 2013 | 323 | 26.92 |
| 7 | A Compositional Look at the Human Gastrointestinal Microbiome and Immune Activation Parameters in HIV Infected Subjects | [Mutlu, EA](https://webofscience.clarivate.cn/wos/author/record/44743715) | 2014 | 287 | 26.09 |
| 8 | Altered Virome and Bacterial Microbiome in Human Immunodeficiency Virus-Associated Acquired Immunodeficiency Syndrome | [Monaco, CL](https://webofscience.clarivate.cn/wos/author/record/15863869) | 2016 | 268 | 29.78 |
| 9 | [Gut Microbiota Linked to Sexual Preference and HIV Infection](https://webofscience.clarivate.cn/wos/woscc/full-record/WOS:000375078200027) | Noguera-Julian, M | 2016 | 264 | 29.33 |
| 10 | Pathogenic Simian Immunodeficiency Virus Infection Is Associated with Expansion of the Enteric Virome | [Handley, SA](https://webofscience.clarivate.cn/wos/author/record/2469623) | 2012 | 221 | 17.00 |
